# Preoperative systemic immune-inflammation index predicts prognosis and guides clinical treatment in patients with non-small cell lung cancer

**DOI:** 10.1042/BSR20200352

**Published:** 2020-03-27

**Authors:** Xue Yan, Guowei Li

**Affiliations:** 1 Department of Respiration Medicine, The First Affiliated Hospital of Jinzhou Medical University, Jinzhou, Liaoning, China; 2 Department of Spine Surgery, The First Affiliated Hospital of Jinzhou Medical University, Jinzhou, Liaoning, China

**Keywords:** adjuvant chemotherapy, non-small cell lung cancer, prognosis, systemic immune-inflammation index

## Abstract

**Objectives:** The purpose of the present study was to evaluate the prognostic value of a systemic immune-inflammation index (SII) and the relationship between SII and the effectiveness of postoperative treatment in patients with non-small cell lung cancer (NSCLC).

**Methods:** A total of 538 patients diagnosed with NSCLC who had undergone curative surgery were retrospectively enrolled in the study. Clinicopathologic and laboratory variables were collected. SII was defined as neutrophil × platelet/lymphocyte counts. Both univariate and multivariate analyses were performed to analyze the prognostic value of these factors.

**Results:** The preoperative SII level was associated with sex, smoking history, histological type, lesion type, resection type, pathological stage, neutrophil/lymphocyte ratio (NLR), platelet/lymphocyte ratio (PLR), lymphocyte/monocyte ratio (LMR), fibrinogen and bone metastasis (*P*<0.05). The univariate and multivariate analyses revealed that SII was an independent prognostic factor for disease-free survival (DFS, *P*=0.033) and overall survival (OS, *P*=0.020). Furthermore, the prognostic value of SII was also verified regardless of the histological type and pathological stage. The subgroup analysis demonstrated that patients with a high SII may benefit from adjuvant therapy (*P*=0.024 for DFS and *P*=0.012 for OS).

**Conclusion:** An increased preoperative SII may independently predict the poor DFS and OS in patients with resectable NSCLC. SII may help select NSCLC patients who might benefit from adjuvant chemotherapy.

## Background

Lung cancer is the most common type of cancer worldwide and the incidence increases 26% annually [1]. The prognosis of non-small cell lung cancer (NSCLC), which accounts for approximately 85% of all lung cancer cases, remains extremely poor [2]. To date, curative surgical resection and adjuvant chemotherapy are recommended as the standard of care for operable patients [3,4]. Unfortunately, patients at each stage of NSCLC have biological heterogeneity, high risk manifested resistance to chemotherapy, early recurrence after surgery and poor outcomes. Therefore, finding one reliable factor that can identify patients at high risk and with resistance to chemotherapy is crucial to treatment selection and improving the prognosis.

In recent years, more attention has been given to genomic tools that explore biomarkers associated with prognosis prediction and targeted treatment [5,6]. However, these genomic technologies are expensive at present and more genomic information needs to be integrated due to the biological complexity of NSCLC [7]. Recently, systemic inflammatory response indicators, such as peripheral neutrophils, lymphocytes and platelets, play important roles in the progression and prognosis of various cancers, including lung cancer [8,9], breast cancer [10] and gastric cancer [11]. Moreover, these peripheral markers can be detected with an easy, convenient method, and its application in prognosis prediction deserves attention. These inflammatory indicators are expressed by inflammatory scores, such as neutrophil/lymphocyte ratio (NLR) and platelet/lymphocyte ratio (PLR), and have been reported to be associated with the prognosis of patients with malignant tumors [8,10,11]. Recently, a new inflammatory index, the systemic immune-inflammation index (SII), defined as neutrophil × platelet/lymphocyte, integrates three inflammatory cells and has been shown to be promising [12]. The prognostic value of SII has been confirmed in various cancers, including NSCLC [13], small cell lung cancer [14], esophageal squamous cell carcinoma (SqCC) [15], breast cancer [16] and hepatocellular cancer [17]. However, to the best of our knowledge, the association of SII with tumor progression and the implication of SII in postoperative adjuvant chemotherapy in operable NSCLC patients remain largely unknown.

Therefore, we conducted the present study to evaluate the prognostic value of SII in patients who had the NSCLC surgically removed. Furthermore, we explored how SII could guide postoperative adjuvant chemotherapy.

## Methods

### Patients

Patients who were diagnosed with resectable NSCLC and hospitalized at the First Affiliated Hospital of Jinzhou Medical University between January 2009 and December 2011 were enrolled in the present study. The inclusion criteria included the following: (1) histologically confirmed NSCLC, (2) complete pulmonary resection and systematic node dissection of the hilar and mediastinal lymph nodes and (3) complete clinical information, laboratory and follow-up data. The exclusion criteria included the following: (1) patients with R1/R2 resection, (2) patients with advanced disease (e.g., malignant pleural effusion/involvement or distant metastasis), (3) patients who received neoadjuvent chemotherapy or radiotherapy, (4) patients who died during the perioperative period and (5) patients with clinical evidence of infection, chronic inflammatory, hematological or autoimmune disease. Finally, a total of 538 cases who matched our inclusion and exclusion criteria were enrolled in the present study. The clinicopathological data were collected from the patients’ medical records. The tumor node metastasis (TNM) stage was assessed based on the 7th edition of the American Joint Committee on Cancer (AJCC) staging manual [18]. This study was approved by the ethics committee of the First Affiliated Hospital of Jinzhou Medical University. All patients signed the informed consent.

### Definition of SII and other variables

Venous blood samplings were taken from all patients within the week prior to surgery and collected in EDTA-containing tubes. We collected the routine laboratory blood measurements, including absolute counts of neutrophils, lymphocytes, monocytes and platelets. The definitions of NLR, PLR, lymphocyte/monocyte ratio (LMR) and SII are calculated using the following equations: NLR = neutrophil count/lymphocyte count; PLR = platelet count/lymphocyte count; LMR = lymphocyte count/monocyte count; SII = platelet count × neutrophil count/lymphocyte count.

### Follow-up

After t he surgery, all patients were followed up every 3 months for the first and second years, every 6 months for the third to fifth years, and then annually thereafter until death or the last follow-up. The primary end point was disease-free survival (DFS), and the secondary end point was overall survival (OS). DFS was defined as the duration of time between the date of surgery and the date of the first recurrence of a tumor and/or distant metastasis or the last follow-up. OS was defined as the duration of time from the date of the surgery to the date of death due to any cause or the last follow-up. The follow-up ended in December 2017.

### Statistical analysis

We constructed receiver operating characteristic (ROC) curves to determine the optional cut-off values of NLR, PLR, LMR, SII and fibrinogen. The values of maximum joint sensitivity and specificity on the ROC plot were defined as the recommended cut-off value. The area under the curve (AUC) was used to assess the predictive value of these markers. A chi-square test was used to assess the association between SII and clinicopathological variables. The Kaplan–Meier method was performed to generate survival curves and the log-rank test was performed to compare the survival rates between the two groups. Hazard ratios (HR) and 95% confidence intervals (CI) were calculated using univariate and multivariate analyses with the Cox proportional hazards model. All statistical analyses were performed using SPSS 17.0 statistical software (SPSS Inc., Chicago, U.S.A.). All *P-*values were bilaterally distributed and *P*<0.05 was considered statistically significant.

## Results

### Patients’ characteristics

A total of 538 patients with histologically confirmed NSCLC were involved in the present study; 343 (63.8%) patients were males and 195 (36.2%) patients were females, with a median age of 60 years (range: 24–82). According to the 7th edition of the TNM staging system, 230 patients were at stage I, 114 patients were at stage II and 194 patients were at stage IIIA. There were 460 (85.5%) patients who underwent a lobectomy and 78 (14.5%) patients who underwent a pneumonectomy. Patients with the histological type of SqCC, adenocarcinoma and other types accounted for 47.2, 40.1 and 12.7%, respectively.

According to the ROC curves, the optional cut-off value of SII was 402.37 with a maximum joint sensitivity of 74.4% and specificity of 51.5%, and the corresponding AUC was 0.598 (95% CI: 0.549–0.646). Similarly, the optional cut-off values of NLR, PLR, LMR and fibrinogen were calculated as 2.35, 150.95, 3.71 and 3.61, respectively. Consequently, the patients were classified into two groups of low or high NLR, PLR, LMR, SII and fibrinogen levels based on the corresponding cut-off values. Three hundred nineteen patients had NLR < 2.35, 219 patients had NLR ≥ 2.35; 351 patients had PLR < 150.95, 187 patients had PLR ≥ 150.95; 287 patients had LMR ≥ 3.71, 251 patients had LMR < 3.71; 199 patients had SII < 402.37, 339 patients had SII ≥ 402.37; 256 patients had fibrinogen < 3.61, 282 patients had fibrinogen ≥ 3.61.

### Correlation between SII and clinicopathological variables in NSCLC patients

The correlations between SII and clinicopathological variables are shown in Table 1. Our results showed that SII was significantly associated with sex (*P*<0.001), smoking history (*P*=0.023), histological type (*P*<0.001), lesion type (*P*<0.001), resection type (*P*<0.001), pathological stage (*P*<0.001), NLR (*P*<0.001), PLR (*P*<0.001), LMR (*P*<0.001), fibrinogen (*P*<0.001) and bone metastasis (*P*=0.007). However, preoperative SII displayed no association with age, tumor location and lymph node metastasis (*P*>0.05).

**Table 1:** The relationships between SII and clinicopathological variables

Variables	Cases	Preoperative SII		*x^2^*	*P*-value
		Low	High		
Sex				19.667	<0.001*
Male	343	103 (30.0%)	240 (70.0%)		
Female	195	96 (49.2%)	99 (50.8%)		
Age (years)				0.646	0.422
≤60	269	95 (35.3%)	174 (64.7%)		
>60	269	104 (38.7%)	165 (61.3%)		
Smoking history				5.183	0.023*
Yes	344	115 (33.4%)	229 (66.6%)		
None	194	84 (43.3%)	110 (56.7%)		
Histological type				22.293	<0.001*
SqCC	254	70 (27.6%)	184 (72.4%)		
Adenocarcinoma	216	105 (48.6%)	111 (51.4%)		
Others	68	24 (35.3%)	44 (64.7%)		
Tumor location				0.406	0.524
Left	223	86 (38.6%)	137 (61.4%)		
Right	315	113 (35.9%)	202 (64.1%)		
Lesion type				12.617	<0.001*
Peripheral	378	158 (41.8%)	220 (58.2%)		
Central	160	41 (25.6%)	119 (74.4%)		
Resection type				18.269	<0.001*
Lobectomy	460	187 (40.7%)	273 (59.3%)		
Pneumonectomy	78	12 (15.4%)	66 (84.6%)		
Lymph node metastasis				1.217	0.270
No	297	116 (39.1%)	181 (60.9%)		
Yes	241	83 (34.4%)	158 (65.6%)		
Pathological stage				20.561	<0.001*
I	230	107 (46.5%)	123 (53.5%)		
II	114	25 (21.9%)	89 (78.1%)		
IIIA	194	67 (34.5%)	127 (65.5%)		
NLR				92.833	<0.001*
Low	319	171 (53.6%)	148 (46.4%)		
High	219	28 (12.8%)	191 (87.2%)		
PLR				68.609	<0.001*
Low	351	174 (49.6%)	177 (50.4%)		
High	187	25 (13.4%)	162 (86.6%)		
LMR				73.343	<0.001*
High	287	154 (53.7%)	133 (46.3%)		
Low	251	45 (17.9%)	206 (82.1%)		
Fibrinogen				44.506	<0.001*
Low	256	132 (51.6%)	124 (48.4%)		
High	282	67 (23.8%)	215 (76.2%)		
Bone metastasis				7.299	0.007*
None	479	282 (48.5%)	299 (51.5%)		
Yes	57	17 (29.8%)	40 (70.2%)		

* Was considered to be statistically significant.

### Univariate and multivariate survival analyses

**Figure 1:**
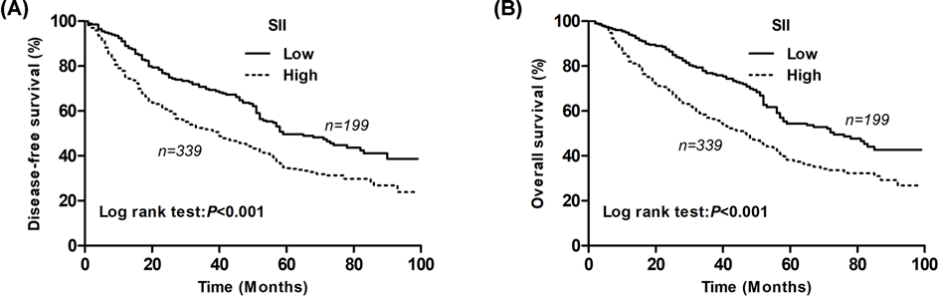
Kaplan–Meier survival curves for NSCLC patients according to the SII levels (**A**) The 5-year DFS rate of patients in the low SII group was significantly higher than those in the high SII group (49.7 vs. 34.9%, *P*<0.001). (**B**) The 5-year OS rate of patients in the low SII group was significantly higher than those in the high SII group (54.3 vs. 38.2%, *P*<0.001).

The median follow-up time for all the patients was 54.0 (range: 3–108) months. The cumulative 5-year DFS and OS rates were 40.4 and 44.1%, respectively. The Kaplan–Meier analysis showed that the low SII group had a markedly higher 5-year DFS rate than the high SII group (49.7 vs. 34.9%; *P*<0.001; Figure 1A). Furthermore, the low-SII group had a markedly higher 5-year OS rate than the high-SII group (54.3 vs. 38.2%; *P*<0.001; Figure 1B).

Using a univariate Cox regression analysis, we found that lesion type (*P*=0.015), resection type (*P*=0.031), pathological stage (*P*<0.001), NLR (*P*=0.010), PLR (*P*=0.026), LMR (*P*<0.001), SII (*P*<0.001) and fibrinogen (*P*<0.001) were significantly associated with DFS. Furthermore, age (*P*=0.018), smoking history (*P*=0.042), lesion type (*P*=0.003), resection type (*P*=0.007), pathological stage (*P*<0.001), NLR (*P*=0.008), PLR (*P*=0.020), LMR (*P*<0.001) SII (*P*<0.001) and fibrinogen (*P*<0.001) were significantly associated with OS (Table 2).

**Table 2:** Univariate analysis of DFS and OS for all NSCLC patients

Variable	DFS			OS		
	HR	95% CI	*P*	HR	95% CI	*P*
Age (≤60/>60)	0.838	0.677–1.038	0.106	0.768	0.618–0.955	0.018*
Sex (male/female)	1.166	0.931–1.459	0.181	1.234	0.981–1.552	0.072
Smoking history (yes/none)	1.148	0.917–1.437	0.229	1.271	1.009–1.601	0.042*
Histological subtype (SqCC/non-SqCC)	1.145	0.923–1.419	0.218	1.085	0.873–1.350	0.461
Tumor location (left/right)	0.873	0.702–1.085	0.221	0.851	0.681–1.062	0.154
Lesion type (central/peripheral)	1.331	1.058–1.673	0.015*	1.422	1.128–1.793	0.003*
Resection type (lobectomy/pneumonectomy)	1.381	1.029–1.852	0.031*	1.493	1.115–2.000	0.007*
Pathological stage (IIIA/I, II)	1.594	1.407–1.804	<0.001*	1.660	1.462–1.884	<0.001*
NLR (high/low)	1.327	1.070–1.647	0.010*	1.343	1.079–1.673	0.008*
PLR (high/low)	1.287	1.031–1.608	0.026*	1.307	1.043–1.639	0.020*
LMR (high/low)	0.648	0.523–0.803	<0.001*	0.606	0.487–0.753	<0.001*
SII (high/low)	1.584	1.259–1.993	<0.001*	1.662	1.314–2.101	<0.001*
Fibrinogen (high/low)	1.476	1.189–1.831	<0.001*	1.644	1.319–2.051	<0.001*

* Was considered to be statistically significant.

The clinicopathological variables that were found to be significant (*P*<0.05) with the univariate analysis were further investigated in a multivariate analysis. Our results showed that pathological stage (*P*<0.001), LMR (*P*=0.042) and SII (*P*=0.033) were independent prognostic factors associated with DFS. Furthermore, age (*P*=0.004), pathological stage (*P*<0.001), LMR (*P*=0.017), SII (*P*=0.020) and fibrinogen (*P*=0.042) were independent prognostic factors associated with OS (Table 3).

**Table 3:** Multivariate analysis of DFS and OS for all NSCLC patients

Variable	DFS			OS		
	HR	95% CI	*P*	HR	95% CI	*P*
Age (≤60 vs. >60)	–	–	–	0.719	0.576–0.899	0.004*
Smoking history (yes/none)	–	–	–	1.051	0.824–1.342	0.688
Lesion type (central/peripheral)	1.082	0.840–1.393	0.541	1.111	0.858–1.439	0.425
Resection type (lobectomy/pneumonectomy)	1.083	0.789–1.488	0.620	1.172	0.852–1.613	0.328
Pathological stage (IIIA/I, II)	1.558	1.370–1.771	<0.001*	1.631	1.431–1.860	<0.001*
NLR (high/low)	1.009	0.785–1.296	0.944	1.028	0.796–1.326	0.835
PLR (high/low)	1.114	0.869–1.427	0.394	1.110	0.865–1.423	0.412
LMR (high/low)	0.785	0.621–0.991	0.042*	0.745	0.585–0.949	0.017*
SII (high/low)	1.338	1.024–1.750	0.033*	1.386	1.053–1.823	0.020*
Fibrinogen (high/low)	1.221	0.971–1.537	0.088	1.279	1.008–1.621	0.042*

* Was considered to be statistically significant.

### Subgroup analysis for the prognostic value of SII

**Figure 2:**
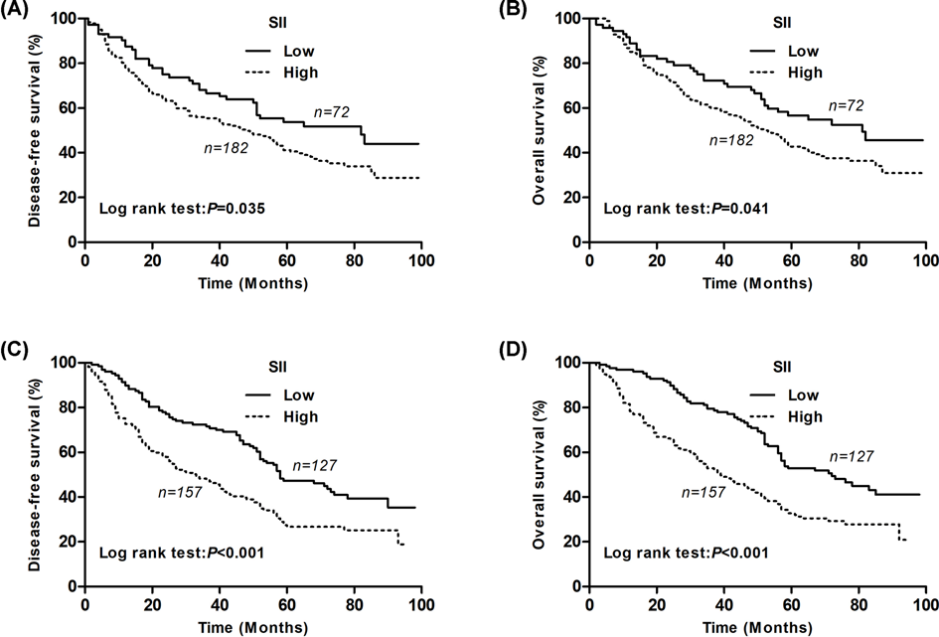
Kaplan–Meier survival curves for SqCC and non-SqCC patients according to the SII levels (**A**) Kaplan–Meier survival curve of DFS for SqCC patients (log-rank, *P*=0.035). (**B**) Kaplan–Meier survival curve of OS for SqCC patients (log-rank, *P*=0.041). (**C**) Kaplan–Meier survival curve of DFS for non-SqCC patients (log-rank, *P*<0.001). (**D**) Kaplan–Meier survival curve of OS for non-SqCC patients (log-rank, *P*<0.001).

For patients with SqCC, the 5-year DFS rates in the SII-low and SII-high groups differed significantly and were 53.7 and 41.2%, respectively (*P*=0.035, Figure 2A). The 5-year OS rates in the SII-low and SII-high groups were 56.7 and 42.8%, respectively (*P*=0.041, Figure 2B). For the non-SqCC patients, the 5-year DFS rates in the SII-low and SII-high groups differed significantly and were 47.3 and 27.5%, respectively (*P*<0.001, Figure 2C). The 5-year OS rates in the SII-low and SII-high groups were 53.0 and 32.8%, respectively (*P*<0.001, Figure 2D).

**Figure 3:**
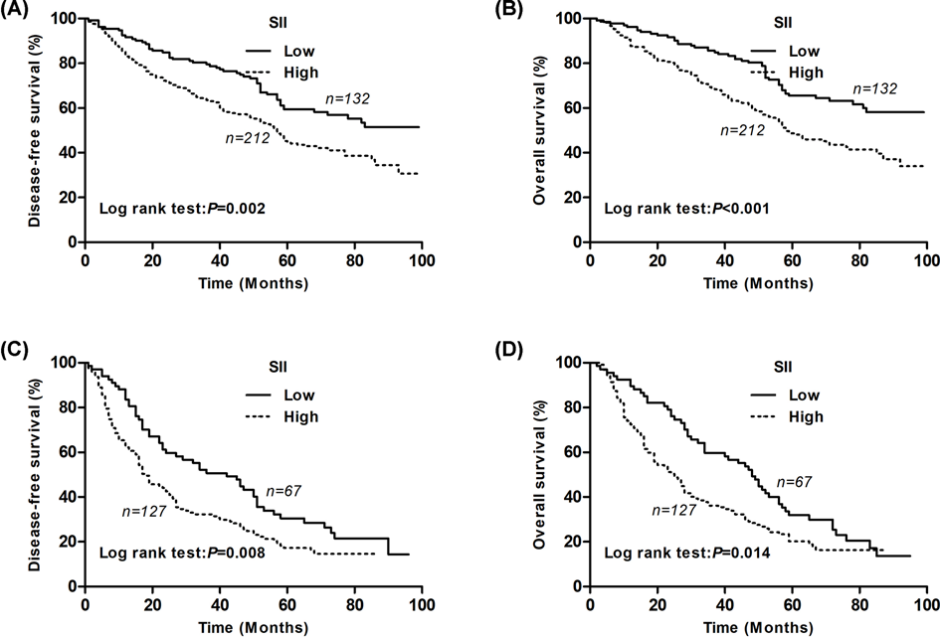
Kaplan–Meier survival curves for stages I, II and IIIA NSCLC patients according to the SII levels (**A**) Kaplan–Meier survival curve of DFS for stage I, II NSCLC patients (log-rank, *P*=0.002). (**B**) Kaplan–Meier survival curve of OS for stage I, II NSCLC patients (log-rank, *P*<0.001). (**C**) Kaplan–Meier survival curve of DFS for stage III NSCLC patients (log-rank, *P*=0.008). (**D**) Kaplan–Meier survival curve of OS for stage III NSCLC patients (log-rank, *P*=0.014).

For patients with cancer stages I–II, the Kaplan–Meier analysis and log-rank test showed significant differences in DFS and OS among the two SII groups (*P=*0.002 for DFS and *P*<0.001 for OS) (Figure 3A,B). For patients with cancer stage IIIA, the Kaplan–Meier analysis and log-rank test showed significant differences in DFS and OS among the two SII groups (*P=*0.008 for DFS and *P=*0.014 for OS) (Figure 3C,D).

### Clinical utility of SII for selection of postoperative adjuvant therapy in NSCLC patients

Our results from the univariate and multivariate survival analyses showed that SII had the potential ability to provide prognostic information. We also conducted a subgroup analysis to explore whether SII had the potential ability to identify patients who would benefit from adjuvant chemotherapy after surgical resection. In our study, 228 (42.4%) patients underwent complete surgical resection only and 310 (57.6%) patients underwent surgery and adjuvant chemotherapy.

**Figure 4:**
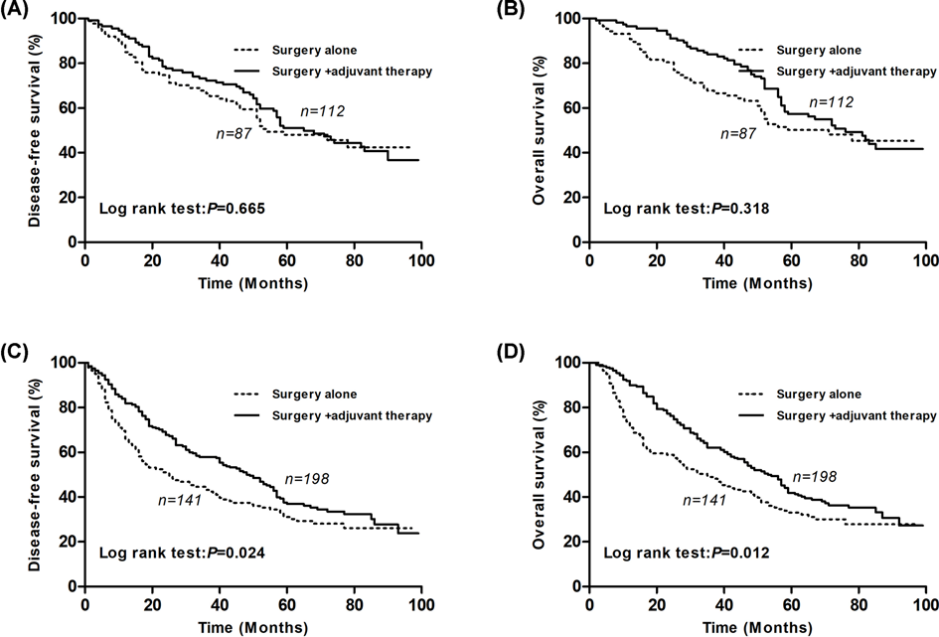
Kaplan–Meier survival curves between the surgery plus chemotherapy group and the surgery alone group for NSCLC patients based on the SII levels (**A**) Kaplan–Meier survival curve of DFS for low SII patients (log-rank, *P*=0.665). (**B**) Kaplan–Meier survival curve of OS for low SII NSCLC patients (log-rank, *P*=0.318). (**C**) Kaplan–Meier survival curve of DFS for high SII NSCLC patients (log-rank, *P*=0.024). (**D**) Kaplan–Meier survival curve of OS for high SII NSCLC patients (log-rank, *P*=0.012).

In the subgroup of patients with low SII, there was no difference for both the DFS and OS (*P*=0.665 for DFS and *P*=0.318 for OS; Figure 4A,B). However, in the subgroup of patients with high SII, our results revealed that the patients who underwent surgery and adjuvant chemotherapy had a better DFS and OS (*P*=0.024 for DFS and *P*=0.012 for OS; Figure 4C,D). SII may be useful for selecting patients who may benefit from adjuvant chemotherapy.

## Discussion

Optimal NSCLC treatment decisions could improve prognosis, which requires a reliable prognosis evaluation. The development of a prognosis prediction tool will help evaluate the prognosis and select high-risk patients who could benefit from surgery or chemotherapy. Although the TNM staging is the standard for prognosis evaluation, preoperative prediction remains difficult and is not sufficient for an accurate survival evaluation [19]. In this study, we validated that SII can be used as a systemic inflammation response index to evaluate the prognosis and to guide treatment in patients with resectable NSCLC.

The association between chronic inflammation and cancer was first reported in 1909 [20]. Over the past decades, a growing amount of research has revealed that systemic inflammation is correlated with proliferation, invasion and metastasis of tumor cells in different cancer types [21,22]. Systemic inflammation could be monitored by using peripheral hematologic markers, such as neutrophils, leukocytes and platelets. Based on the relationship between inflammation and cancer, in recent years, inflammatory marker-based scores, such as NLR and PLR have been reported to be associated with the prognosis of patients with various cancers, including lung cancer [8], breast cancer [10,23], gastric cancer [11,24] and colorectal cancer [25]. However, NLR and PLR only include two types of cells. If these three factors were integrated into one index, it would provide a better predictive score. SII, initially reported by Hu et al. [26], was defined as neutrophil × platelet/lymphocyte and seemed to be a stronger prognostic predictor. Indeed, Guo et al. [27] demonstrated that SII may have a better prognostic ability when compared with NLR and PLR in patients with NSCLC. In the present study, we focused on the prognostic value of SII in patients with NSCLC. Our results showed that an elevated preoperative SII was correlated with progressive biological behavior, including bone metastasis. In the further univariate and multivariate analyses, preoperative SII was an independent prognostic factor for OS in patients with surgically resectable NSCLC. These results were consistent with previous investigations on NSCLC [13,27]. Furthermore, our study was the first to analyze the relationship between preoperative SII and DFS in patients with surgically resectable NSCLC. Our results showed that an increased preoperative SII independently predicted a shorter DFS in patients with NSCLC. SII still retained prognostic significance regardless of the histological type and TNM stage subgroups. Therefore, NSCLC patients with a high preoperative SII score seemed to have a poorer prognosis when compared with patients with a low SII score.

For patients with completely resectable NSCLC, adjuvant chemotherapy has been shown to improve survival [28]. However, NSCLC patients with the same TNM stage who underwent the same treatment regimen may have a different prognosis. Therefore, selecting the optimal treatment for patients may help improve their clinical outcomes. However, so far, none of the biomarkers in NSCLC have been incorporated into treatment management. In the present study, all NSCLC patients were divided into low- and high-risk groups based on the SII score. Our results demonstrated that NSCLC patients with high SII may be more likely to benefit from postoperative adjuvant therapy. However, large prospective and randomized controlled studies are needed to confirm our results in the future.

Our study has some novelties. First, the study demonstrated that NSCLC patients with a high preoperative SII score not only had a shorter DFS but also had a worse OS. Preoperative SII could help prognosis evaluation and risk stratification. Second, SII is clinically useful for selecting NSCLC patients who may benefit from adjuvant therapy. Finally, SII, which is based on the available and non-expensive peripheral hematologic biomarkers in routine daily practice, might be applied to clinical practice in the future. Despite the novelty of our study, there are some limitations of our research. First, the study was a single-center retrospective study, therefore, selection and analytical biases are inevitable. Second, the SII score was based on the peripheral blood cell analysis, which is easily affected by factors such as hypercholesterolemia, infection and blood circulation capacity. Third, the cut-off value of SII in the current study was decided using an ROC curve, which may be an arbitrary value. Currently, there is no uniform cut-off value for SII, and different research subjects may obtain different cut-off values. Therefore, in the future, prospective and multicenter studies will be needed to confirm our preliminary results.

In summary, preoperative SII based on the standard laboratory measurements is related to postoperative DFS and OS and could be an independent prognostic predictor in patients with surgically resectable NSCLC. Moreover, preoperative SII could help clinicians select patients who could benefit from adjuvant chemotherapy and improve their clinical outcomes. Therefore, this simple and economic inflammation-based indicator may serve as a useful biomarker for prognosis prediction and planning therapeutic strategies for patients with resectable NSCLC in the future.

## Abbreviations

AUC: area under the curve
CI: confidence interval
DFS: disease-free survival
LMR: lymphocyte/monocyte ratio
NLR: neutrophil/lymphocyte ratio
NSCLC: non-small cell lung cancer
OS: overall survival
PLR: platelet/lymphocyte ratio
SII: system immune-inflammation index
SqCC: squamous cell carcinoma
TNM: tumor node metastasis
